# Potential of a suite of robot/computer-assisted motivating systems for personalized, home-based, stroke rehabilitation

**DOI:** 10.1186/1743-0003-4-6

**Published:** 2007-03-01

**Authors:** Michelle J Johnson, Xin Feng, Laura M Johnson, Jack M Winters

**Affiliations:** 1 Medical College of Wisconsin, Dept. of Physical Medicine & Rehabilitation, 9200 W. Wisconsin Ave, Milwaukee, WI 53226, USA; 2 Marquette University, Dept. of Biomedical Engineering, Olin Engineering Center, Milwaukee, WI, USA; 3 Clement J. Zablocki VA, Dept. of Physical Medicine & Rehabilitation, Rehabilitation Robotics Research and Design Lab, 5000 National Ave, Milwaukee, WI, USA

## Abstract

**Background:**

There is a need to improve semi-autonomous stroke therapy in home environments often characterized by low supervision of clinical experts and low extrinsic motivation. Our distributed device approach to this problem consists of an integrated suite of low-cost robotic/computer-assistive technologies driven by a novel universal access software framework called UniTherapy. Our design strategy for personalizing the therapy, providing extrinsic motivation and outcome assessment is presented and evaluated.

**Methods:**

Three studies were conducted to evaluate the potential of the suite. A conventional force-reflecting joystick, a modified joystick therapy platform (TheraJoy), and a steering wheel platform (TheraDrive) were tested separately with the UniTherapy software. Stroke subjects with hemiparesis and able-bodied subjects completed tracking activities with the devices in different positions. We quantify motor performance across subject groups and across device platforms and muscle activation across devices at two positions in the arm workspace.

**Results:**

Trends in the assessment metrics were consistent across devices with able-bodied and high functioning strokes subjects being significantly more accurate and quicker in their motor performance than low functioning subjects. Muscle activation patterns were different for shoulder and elbow across different devices and locations.

**Conclusion:**

The Robot/CAMR suite has potential for stroke rehabilitation. By manipulating hardware and software variables, we can create personalized therapy environments that engage patients, address their therapy need, and track their progress. A larger longitudinal study is still needed to evaluate these systems in under-supervised environments such as the home.

## Background

Stroke-induced impairments and disabilities, especially those affecting the upper extremity, often disrupt a person's ability to function independently in his or her chosen living environment [1]. Rehabilitation training of the impaired upper extremity focuses on reducing impairment and improving independent function on various daily activities (ADLs) salient to patients' real-life environments [1-4]. It is considered effective and successful if patients are able to transfer motor and functional gains seen during supervised therapy to their living environments, i.e., they are able to use their impaired arm away from therapist supervision [2-4].

Most stroke therapy environments for the upper arm, including robot-assisted ones, are not able to consistently demonstrate carryover of motor gains during upper extremity training to increased functional use of the impaired arm in under-supervised environments [5,6]. Robotic-assisted therapy devices provide autonomous training where patients can engage in repeated and intense practice of goal-directed tasks leading to improvements in motor function [7-10]. Results of clinical trials using these systems are positive, and motor gains seen and captured by sensitive kinematic variables such as movement smoothness and movement time correlate well to clinical motor impairment scales such as the Fugl-Meyer [11] but not as well to functional ones [5].

While encouraged by the success by these approaches, there is also a need to improve the cost-to-benefit ratio of robot-assisted therapy strategies and their effectiveness in extending motor gains to ADLs and increasing the functional use of the impaired arm. These goals are challenging when considered in the context of providing autonomous stroke therapy for environments characterized by the low supervision by clinical experts, less intensive training, low extrinsic motivation, subjective assessment of outcomes, etc [4,12]. In addition, semi-autonomous training emphasizes the issues of timely monitoring and of the usability and accessibility of the system [13].

The vision of the combined Falk Neurorehabilitation Engineering Research Lab and the Rehabilitation Robotics Research and Design Lab (RRRD) for meeting these needs combines robotic therapy and tele-rehabilitation technologies with motivating rehabilitation strategies. We created an upper arm stroke therapy suite consisting of several affordable hardware platforms and a novel and customizable universal software platform. The hardware platforms include commercial force-reflecting joysticks and wheels with the custom-made platforms are UniTherapy [14], TheraDrive [15], and TheraJoy [16]. The hardware and software platforms are reconfigurable and can promote unilateral or bilateral arm movements. The nature of the UniTherapy software is such that we can expand our hardware suite to accommodate other customized and commercial hardware systems that use the gaming device port. We use a distributed framework that supports remote interactions with therapists and game-based activities for therapy and assessment. These combined systems are our low-cost, robotic and computer-assisted motivating rehabilitation (Robot/CAMR) suite.

This paper will outline our design approach as well as provide evidence for its potential usefulness in stroke rehabilitation. First, we discuss our *design strategy *for *personalizing *the therapy protocol and user interface, for sustaining *motivation *to engage in therapy, and for providing objective *assessment *of the tailored protocol and its outcomes. Second, we discuss example results from three experiments that were conducted to evaluate the potential of our software and hardware suite for creating versatile therapy environments. We focused on evaluating several devices and device settings (e.g., device location) to determine their influence on performance outcomes and to distinguish across persons at different functioning levels. Our conclusions suggest that the Robot/CAMR suite has potential for stroke rehabilitation and by manipulating hardware and software variables we can create therapy that will meet patients' therapeutic needs and potentially engage them.

### Design strategies

#### Design strategy for personalizing interfaces and protocols

Each potential patient or client has different abilities, functional needs and interests. This suggests that personalization of a prescribed therapeutic program makes sense. An emphasis on more autonomous use of robotic therapy systems makes personalization of the human-technology interface very important. There are two key components of personalized interfaces: the physical interface (e.g., the device itself, its physical settings, and range of operation of the device relative to the user's torso) and the communication interface (e.g., software and monitor, including software support for possible alternative interface features). Each is briefly discussed.

The physical interface for most existing robotic applications consists of a single handle (or wrist cuff) that is coupled to a multi-link manipulator, in some cases with a form of passive antigravity support. Such a manipulator facilitates use of the handle/cuff within different regions of the workspace, ideally spanning a three-dimensional (3D) space [5-10]. Our alternative strategy is to offer a suite of 1- and 2- degrees of freedom (DOF) low-cost physical interfaces, with each additionally able to be mounted in different parts of the arm workspace. A natural thought is that these simple devices would limit the options for therapy. However, inspection of the tasks employed by the high-end robotic systems [6] indicates that they tend not to take full advantage of the complex capabilities of these advanced robotic systems, but rather focus on using a limited subset of the arm workspace. In addition, the mechanical limitations of similar systems may be outweighed by cost reduction.

Perhaps the greater research challenge relates to what and how to personalize. In conventional therapy, therapists routinely customize and adjust the focus of therapeutic intervention, especially as a client demonstrates improvement. This suggests the importance of a training protocol that is easily (and often purposefully) varied, both in terms of use of the full "ability" workspace (including force assistance to gently expand this ability space) and of the types of tasks performed within the workspace.

There has been limited focus in stroke rehabilitation on the accessibility and personalizing of the communication interface. This may have been due to the heavy assumptions that the stroke therapy interface is not controlled by the impaired user. The literature from mobile, wheelchair and workstation rehabilitation robotics can help inform this process [17-20]. In these examples, the interface is customized for the user's expertise level (e.g., novel, expert, and engineer), for their disability level (e.g., voice control if speech is difficult), and for the task execution level (e.g., autonomous or semi-autonomous).

**Figure 1:**
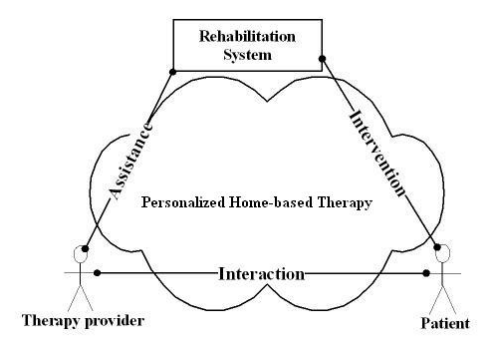
**Personalized Therapy Interactions**. Use Cases of Personalized Rehabilitation System under Home-based Therapy context: Rehabilitation system provides goal-directed assessment and therapeutic intervention to patient; therapy providers interacted with patients and observe their performance; based on the observation, therapy providers optimize their therapy plan with the assistance by rehabilitation system.

In our approach, the UniTherapy platform [21] was designed to permit the personalization of the therapy via tasks, devices, and tele-support of the relationships between patient, therapy provider and the rehabilitation technology (shown in Fig. 1). The following outlines these relationships:

##### • Rehabilitation system to therapy provider interface

Therapy providers can design "tailored" goal-directed assessment or fun tasks for their patient based on their capability and can later update the tasks based on the progress Design templates allow the therapy provider to design individual tasks. A utility called "task design wizard" provides questions to aid in the design of simple tasks. This allows the therapy provider to participate in the rehabilitation process more actively. Complementing the tasks, therapists can also choose from a battery of devices and device settings to complete the intervention protocol.

##### • Patient to rehabilitation system interface

UniTherapy supports therapeutic devices ranging from standard force-feedback joystick, or driving wheels to customized third-party devices such as TheraDrive and TheraJoy discussed in subsequent sections, with the goal-directed task being able to be mapped between a subject's capability space and device workspace so that most tasks can be guaranteed to be accomplished. Compliant with ANSI INCITS 389–393 standard [22], it allows user to interact with the system by personal assistance device (e.g., PocketPC) with user interfaces to be generated automatically based on user preferences and capabilities [23].

##### • Patient to therapy provider interface

By integrating tele-conference capabilities, therapy providers can observe the patient performance remotely and interact with patients by audio, video, and text messages and thus a therapy provider can adjust the intervention protocols based on observation with the hypothesis that more frequent and timely assessments will optimize the intervention outcome.

In this paper, we focus on examining how the hardware and software variables we have implemented in the suite such as the device type and device settings influence subject performance.

#### Design strategy for sustaining motivation

A key aspect to personalizing therapy is considering how subject's interests can be incorporated into the therapy to improve task relevance, purposefulness and extrinsic motivation to stay engaged in the therapy. This design strategy addresses the need for sustaining motivation to use the impaired arm in under-supervised environments.

Wolf, Taub and others showed that stroke survivors often have diminished spontaneous use of their impaired arm in real world tasks and a learned bias for use of their less-affected arm [24,25]. A brief review of the literature indicates that non use of the impaired arm may occur because of one or more scenarios (Table 1) [1-4,24-27].

**Table 1:** Summary of common scenarios leading to decreased impaired arm involvement during real life

	GENERAL CASES	SCENARIOS
1	The immediate rewards of engaging in compensatory behaviors are more apparent and achievable than for engaging restorative behaviors	Patient becomes confused and feels encouraged to engage in both compensatory activities and restorative behaviors. Patient becomes satisfied with the level of independence attained either through caregivers (proxy control) or through the compensatory strategies.
2	The effort (or cost) to engage in restorative behaviors is beyond their ability.	Patient stops using the impaired arm due to the frustration encountered during attempts to use the arm. The effort to engage in restorative behavior is prohibitive and therefore achieving bilateral arm use is perceived as an unrealistic goal. Patient perceives that the activities are too challenging and therefore impossible to achieve or too easy and therefore irrelevant. Patient loses range of motion, muscle strength, dexterity and other motor abilities due to factors such as abnormal muscle activation and force generation. Patient loses sensory feedback in the impaired limb. Patient has a frontal lobe lesion and diminished motivation.
3	The effort to engage in restorative behaviors is not seen as resulting in getting their perceived needs met.	Patient perceives that continuing in rehabilitation is unproductive because it will not help in regaining previous roles in life.
4	The reasons (or incentives) given to encourage them to engage in restorative behaviors are not sufficient.	Patient believes their discharge from the hospital signals the end of recovery and believes the standard predictions that there is minimal to no recovery after 6 months. Patient loses the ability to focus on treatment activities because of neurological deficits and must be reminded to do it.

These behaviours clearly indicate that, after stroke rehabilitation, the use of the impaired arm away from the clinic cannot be assumed. The literature offers some suggestions on how to overcome tendencies to not use the impaired arm. For example, Trombly and Ma[4,28] discuss sustaining motivation to use the impaired arm through the use of game-based and purposeful activities (real or virtual) that tap into patients' life roles. Wolf, Taub and colleagues [29] have use of bindings on the less-affected arm combined with intense one-on-one supervision of task practice of ADLs in their forced-use and constraint-induced (CI) therapies. Lum and colleagues via an automated CI environment (AutoCITE) used real tasks and positive feedback [30] to motivate compliance in the under-supervised environment. Bach-y-Rita et al [31] and Reinkensmeyer [32] used games and simple or commercial hardware to assess and motivate arm use.

Our approach also uses commercially available, game-based activities and custom assessment activities along with tele-supported clinical interactions to create an enjoyable therapy. We attempt to tap the competitive desire to win at the games presented and by doing so, we hope to motivate them to become immersed in the game, work harder and use the arm longer. In combination, we use a familiar battery of off-the-shelf technologies for affordability, and modify them so that they can be used within a therapy environment. By doing so, we make the therapy approachable and more like everyday play. While we do not explicitly analyze the effect of this strategy we briefly discuss feedback from our users.

#### Design strategy for assessing functional outcomes

Assessment is another critical component for evaluating human performance so as to support the optimizing of intervention plans, for providing feedback to assist in sustaining motivation, and for providing an alternative therapy environment. The provision of these assessment tools is fundamental to most robot-assisted stroke therapy systems [8,9,33]. The ability to provide an objective assessment of therapeutic outcomes is a feature that therapists require from these systems [34,35]. Assessment metrics have also been used as an online measure to provide performance feedback during or immediately following a task trial. These types of feedback are especially important in semi-autonomous or autonomous training, because they serve as extrinsic motivators for performance. For example, Lum and colleagues [30] display performance means and provide verbal encouragement such as "Wow!" via AutoCITE.

Goal-directed tasks with the affected limb in stroke subjects are typically characterized by decreased range of motion (ROM), movement speed, smoothness, coordination, and abnormal pattern of muscle activation [36]. This suggests that the form of assessment tasks should be varied and be able to be customized to target the individual subject's motor deficit. Our approach via UniTherapy implements four toolboxes consisting of customizable assessment tasks to evaluate different aspects of motor performance to provide timely feedback to optimize intervention plans and commercial games as fun therapy tools to provide encouragement and feedback to sustain motivation. These toolboxes are outlined below:

• The *ROM toolbox *can be used to assess the user's initial and final capability ROM when using an input device and optionally used to map between the input device workspace range and the user's capability range by a 2D transformation algorithm [14].

• The *tracking toolbox *implements discrete tracking and continuous tracking. Discrete tracking requires the subject to move a cursor into a target window as quick as they can and stabilize before the target jumps. Continuous tracking instructs subjects to follow the continuously moving target and minimize the tracking error as much as possible.

• The users' stable motor performance is also evaluated using the *System Identification toolbox*. Predefined force perturbations are applied to the subject under a certain instruction (e.g., "hold," "relax"). The force data and experimenter's instruction are recorded as input while subject's movement data is recorded as output.

• The *Fun toolbox *contains third-party computer game programs that can be integrated into the framework with the system collecting input device signals without affecting the game performance at the front end. A collection of simple arcade games (e.g., several card and poker games, driving games, Pong, Pac-man) are current examples of fun therapy tools being used.

In UniTherapy, a number of customized and standard performance metrics examining accuracy [36-38], smoothness [33], quickness [33,36], stability, motivation [40], strength [39], and so on have been implemented (see Table 2). These metrics were implemented to allow us to assess treatment changes due to the devices and subjects and monitor training intensity and motivation. It is beyond the scope of this paper to evaluate all the metrics implemented in the UniTherapy assessment battery. In this paper, we focus on using proven sensitive metrics such as the Root Mean Square Error (RMSE) for accuracy and the movement speed for quickness to quantify the influence of device type on kinematic performance of able-bodied persons and high and low-to-medium functioning stroke survivors.

**Table 2:** Summary of possible performance metrics that could be used in assessment tasks and fun therapy tool [41]

**Assessment Category**	**Metric Name**	**Definition**	**Remark**
Range of Motion (ROM)	ROM Area Ratio	The ratio of the area size of user capability space to the input device work space.	Reflects the user's ***Movement Range ***in the range [0, 1]; ideally this value should be close to 1.
Discrete Tracking	Reaction Time	The time from the jump of the target to the first significant movement by subject.	Reflects the human machine system response Capability (***Reaction quickness***).
	Movement Time	The time between the end of the reaction time to the time after the human subject stayed within the target stably.	Reflects the ***Movement Quickness***.
	Movement Speed	Movement speed is the average speed within the movement time window.	Reflects the ***Movement Quickness ***in the movement time window.
	Error	The average distance from the target position to the subject position.	Reflects overall performance ***Accuracy***.
	Deviation	The average distance from the subject position to straight target path line.	Reflects ***Movement Curvature***. This metric is for Joystick only.
	Peak Speed Number	The number of peaks in the speed profile within the movement time window.	Fewer *PN *represent fewer periods of acceleration and deceleration, making a more ***Smoothness ***movement.
	Dwelling Percentage Time in Target	The percentage of time subject staying in the target during the dwell window period.	The metric is in the range [0, 1]; ideally this value should be close to 1.The higher value indicates a better ***Stability ***performance.
Continuous Tracking	Percentage Time on Target	The percentage time the human subject staying within the target	Reflects overall performance ***Accuracy ***and ***Stability***.
	Root Mean Square Error	The squared root of the mean-squared distance from subject position to the target position.	Reflects movement ***Accuracy***.
	Average Deviation	The average deviation distance from the subject position to straight target path line.	Reflects ***Movement Curvature***. This metric is for Joystick only.
System Identification	Perturbation Range	The movement range of the human subject in the perturbation direction.	Depends on the instruction to human subject. In case "holding" instruction, the bigger value
	Perturbation Standard Deviation	The standard deviation value of the human subject position in the perturbation direction.	indicates weak ***Strength***; in case "relax" instruction, the bigger value indicates less ***Muscle Stiffness***.
Fun Therapy	ROM Intensity Image	The human subject ROM movement image with the high intensity indicates intensive human movement area.	Reflects ***Movement Range and Intensity ***without overwhelming with movement data when task context is unknown.
	Motivation Score	Used as a multidimensional assessment tool to evaluate subjects' subjective experience related to a target activity in laboratory experiments	Reflects ***Motivation***

## Methods

In this section, we discuss our hypotheses and describe the set-up and protocols used in three separate experiments, which evaluated the Robot/CAMR Suite concept for different sets of hardware systems with the UniTherapy software customized to accommodate 1-dimensional (wheel) and 2-dimensional (joystick) systems.

### Hypotheses

Three study protocols (EP1-EP3) were implemented. Our overall hypothesis is that hardware and software variables implemented in the Robot/CAMR suite influence performance outcomes and thus, provide a useful method for customizing stroke therapy and aiding with therapeutic prescription. Specifically, we examined three hypotheses: **hypothesis 1) **Impairment of human subjects influence performance on goal-directed tasks within and across device types and settings (EP1 and EP2), **hypothesis 2) **Device type influence the kinematic performance of human subjects in goal-directed tasks (EP1 and EP2), and **hypothesis 3) **Device position in the workspace relative to the trunk influence the muscle activation of human subjects in goal-directed tasks (EP3).

### UniTherapy software

We utilize UniTherapy with a Joystick (SideWinder from Microsoft) and wheel force-reflecting technology (Logitech) along with two custom-made therapy platforms, TheraJoy (adapted joystick) and TheraDrive (steering wheel). UniTherapy applied none or varying levels of force-feedback to these devices, depending on the settings and the task; these were derived from a series of force effects such as spring, damper, inertia, constant and so on in DirectX. Position data and force were sampled at 33 Hz.

Spring assistance and resistance force were tested in the EP1 and EP2 studies, with the spring assistance and spring resistance force are defined in equations (1) and (2):

Assistance: *F*_*x*, *y*_= k*(*Subject*_*x*, *y*_- *Target*_*x*, *y*_)     (1)

Resistance: *F*_*x*, *y*_= -k*(*Subject*_*x*, *y*_- *Target*_*x*, *y*_)     (2)

where *F*_*x*,*y *_represents the force at x and y direction, *k *represents the spring coefficient, *Subject*_*x*,*y *_represents the subject position at x and y direction, *Target*_*x*,*y *_represents the target position at x and y direction [41].

The toolboxes in UniTherapy were also customized for each device with a large variety of games that can be customized according to user preferences. The joystick systems used mainly the tracking tasks in rectangular coordinates with both x- and y-directions under the user control. The fun therapy toolbox consisted of third-party games such as solitaire and Pac-man. The wheel systems used both polar and rectangular coordinates for the tracking tasks. The angle of movement and only the x-direction was under user control. The fun toolbox here consisted of two off-the-shelf driving games, SmartDriver and Trackmania.

### Robot/CAMR hardware suite

#### Commercial joysticks and theraJoy

**Figure 2:**
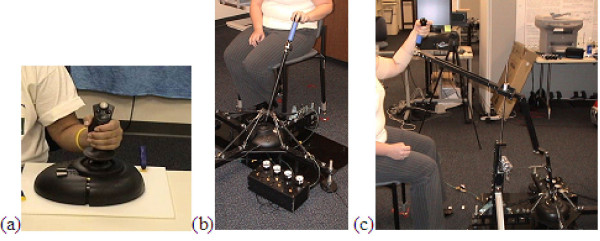
**Joystick Systems**. Conventional Joystick (a) and TheraJoy version 3: Horizontal (bt) and Vertical (c) The vertical linkage system attaches to the horizontal joystick with a ball and socket joint, and a fixed vertical post with a pin and sliding joint

Joystick systems used in studies 1 and 3 (EP1 and EP3) consisted of the TheraJoy and conventional force-feedback joysticks with the UniTherapy software. Figure 2a–c shows the current version of the TheraJoy System along with the conventional joystick.

The TheraJoy system expands the length of a conventional joystick (Microsoft) shaft to nearly one meter with a resting position near the waist of the user. This system incorporates a larger range of motion that can be scaled and modified depending on the anthropometrics and abilities of the user. Pneumatic springs were added to the system to add passive resistance and to compensate for an inverse pendulum effect. A linkage system was added to the extended shaft to incorporate vertical planar motions that are more common to activities of daily living; the system allows vertical movement of the arm expressed as horizontal translation of the joystick. The linkage connects to the shaft of the joystick with a ball and socket joint, and at the sliding shaft with a combination sliding and pin joint. An additional horizontally placed support spring compensates for the effects of gravity and joint friction inherent in the system. The system is accessible to wheelchairs, and patients with varying levels of arm range of motion and hand function.

#### Commercial driving wheels via TheraDrive

**Figure 3:**
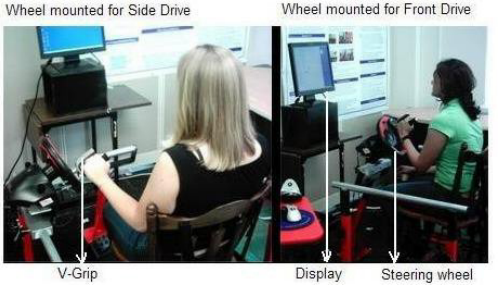
**TheraDrive System for home-based rehabilitation**. This figure shows the driving wheels mounted in front and side configurations with the subject holding onto a v-gripper.

The second study (EP2) was conducted using the TheraDrive interfaced with the UniTherapy software. Figure 3 shows the TheraDrive System in two steering configurations.

TheraDrive is a custom steering environment. One or two force-reflecting wheels (Logitech) can be mounted on the front or side rails of a height-adjustable platform and tilted from 0 to 90 degrees. The platform accommodates wheelchairs and supports front and side unilateral driving and bilateral front steering at any wheel angle. The tilt angle and optional mounting is facilitated by special mounts that uses pin joints to rotate the wheel and tubular clamps to mount wheels to the front or side rails. A special gripper (Mobility Systems) is mounted onto the wheel to ensure the consistent transfer of tangential forces during steering movement. All subjects had to steer while holding onto the gripper. The gripper can be sensorized to measure grip forces and tangential forces during movement.

### Procedures

Experimental protocols EP1 and EP3 involved evaluating the UniTherapy system customized for the conventional joystick and TheraJoy system. These evaluative studies were approved by the Institutional Review Board at Marquette University. Experimental protocol EP2 involved evaluating UniTherapy system customized for the force-feedback steering wheel and the TheraDrive system. This study was approved by the Institutional Review Boards at the Clement J. Zablocki VA and Marquette University.

Sixteen strokes subjects with hemiplegia and twenty able-bodied (Control) subjects participated in these protocols and gave informed consent. Table 3 summarizes the subjects used in each experiment. All stroke survivors were at least six months post-stroke and had been discharged from all forms of physical rehabilitation. All experiments included at least the upper extremity motor control portion of the Fugl-Meyer (UE F-M) assessment test [11] as a tool to assess level of motor impairment of stroke survivors. This test is used to partition stroke survivors into two groups: high function (58–66) and low-to-medium function (22–57).

**Table 3:** Subjects for EP1, EP2 and EP3

Protocol	Subjects Group	Male	Female	Age	UE FM
EP1 (Joysticks)	Able-Bodied	4	4	21–43	N/A
	Stroke-Induced Arm Impairment	3	6	33–76	Low (22–57): 4 High (58–66): 5
EP2 (TheraDrive)	Stroke-Induced Arm Impairment	5	2	55–62	Low (24–56): 3 High (58–66): 4
EP3 (Posture Study)	Able-Bodied	6	6	22–62	N/A

UE FM – Upper Extremity Fugl-Meyer.

#### Joysticks' experimental procedure #1 (EP1) – assessment of performance

This experiment aimed to evaluate the usability of the conventional joysticks and the TheraJoy system with UniTherapy. The experimental protocol consisted of two sessions focusing first on training the individual on using each device (conventional joystick (CJS) and TheraJoy in horizontal (HJS) and vertical (VJS) configurations), then on collecting performance and EMG data on a suite of goal-directed assessment tasks.

In the first session, all joysticks were placed in the position of greatest comfort for the subject, including altered handle position and interface to allow for maximum comfort. Stroke subjects were then evaluated using the ROM toolbox. A test was completed with each of the devices. All subjects then completed several tasks from the Tracking and System Identification toolbox with the conventional joystick. For conventional joystick only, a subset of tasks were then repeated with the horizontal and then vertical TheraJoy. All tasks were repeated with both arms. They completed a game of Solitaire from the Fun Therapy Toolbox using only the conventional joystick. To complete the first day of testing, the subject was introduced to tele-health technology to interact with a remote therapist who loaded the predefined protocol with the UniTherapy software.

On the second day, the tasks were repeated but this time both video and EMG data were also collected. Video data was collected using the Mobile Usability Lab (MU-Lab) [42] and EMG data was collected on eight shoulder and arm muscles (Motion Lab Systems, Inc). Usability surveys were given at the end of the second session to determine the prospective use of the system in the subject's home and their impression of the UniTherapy software and TheraJoy hardware. The questions reported here focused on how subjects enjoyed the device and how easy it was to understand and complete the tasks.

#### Wheels' experimental procedure #2 (EP2) – assessment of performance

The experimental protocol also consisted of two sessions as in EP1, with Day 1 focused on training and Day 2 on collecting a variety of tracking tasks. This study was conducted to evaluate the usability of the TheraDrive system with UniTherapy.

To complete the tracking tasks in both sessions, the wheel was either attached to the front or to the side of the hardware frame and the height was positioned to be comfortable. The wheel was used at a tilt angle of 20 degrees (for normal drive) and 90 degrees (for bus driver mode) (see fig. 3). Subjects held onto the gripper to complete a variety of tracking tasks. The tasks were also completed with or without force-feedback and with either the impaired arm, unimpaired arm, or both. At the end of both days, subjects played the third-party driving games. The UniTherapy program applied spring-like forces to the wheel, which ranged from -100% to 100% of maximum capability. Based on previously derived conversion equations by Johnson et al 2004 [15], the resultant maximum torque was equivalent to 1.850 Nm. Forces were carefully applied so that subjects were able to complete the task at moderate exertion levels.

Surveys were given at the beginning and end of the sessions to determine the prospective use of the system in the subject's home and their impression of the driving games. Specifically, subjects were asked to rate how they enjoyed the device and how easy it was to understand and complete the tasks. Position and video data were collected on both days while EMG data on seven upper arm muscles were only collected only for day 2. Again as in EP1, the EMG and video data are not analyzed here and only representative tracking data are analyzed in the results section.

#### Representative tracking tasks analyzed in EP1 and EP2

The EP1 and EP2 protocols were purposely designed to overlap in a subset of tracking tasks so that human subject performance on various therapeutic interfaces could be compared. The representative results from continuous pseudo-random sinusoidal tracking will be presented here. It is important to note that the joystick tasks required the users to control the motion in TWO directions (both *x *and *y*) while the steering wheel task required the subject to control the task in only ONE direction (*x*) with the y-direction position of the subject automatically set to the y-direction position of the target.

##### Continuous pseudo-random sinusoidal tracking

**Figure 4:**
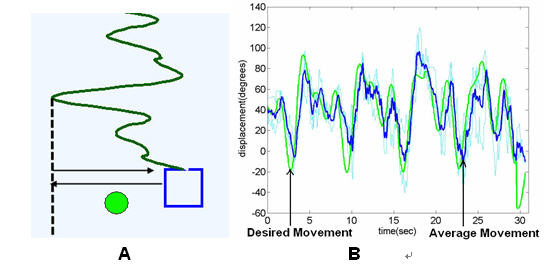
**Representative continuous tracking task**. The screen shot shows the pseudo-random sinusoid task that the subject tried to complete and the average of three trials of a subject from EP2 study when he performed the pseudo-random tracking task and the desired movement.

Subjects in both protocols were asked to complete continuous pseudo-random tracking, which is generated by overlapping three sinusoid curves of various frequencies (1 HZ, 2 HZ and 3 HZ). Subjects were asked to move the wheel or joystick to keep pace with the square box as it moves in a x-direction in a pseudo-random sine pattern; the overlapped sinusoidal curve were shown to human subject as a preview. Figure 4 shows this task along with a representative look at the x-direction motion for the wheel. For the joystick tasks, while human subjects were instructed to control the joystick in both directions to get into the target window, the program only counts x-direction data as success criteria.

##### Pseudo-random target acquisition

Both high and low functional group subjects in both protocols were asked to complete target acquisition tasks where they moved the conventional joystick (EP1) or wheel (EP2) to acquire a the square box with accuracy and at a comfortable speed. The target box was moved to 5 different locations in a pseudo-random pattern, which appears unpredictable to human subjects. Once the subjects get into the target region ("target window"), they received positive visual feedback by a change in color and also a sound cue. They were required to stay as stable as possible for a threshold of success time (defined as "dwell window," DW) for 1 second. After successful completion of DW, the target jumped to the next predefined position.

#### Experimental procedure #3 (EP3) – assessment of postural effects

Each device was anthropometrically positioned in 3–5 locations throughout the arm workspace (i.e. close to the body, far from the body, neutral to the shoulder, neutral to the sternum, etc.). The study was conducted to evaluate the EMG activity of key shoulder and arm muscles and movement paths while using a conventional joystick and the TheraJoy device in both the horizontal and vertical configurations, each within multiple areas of the arm workspace.

For a given device position, two discrete tracking tasks were designed to encompass each device workspace by having the subject track three times in each direction eight points on a rectangle and on a circular starburst, which was characterized by a target centered in a circle of targets at every 45 degrees. Subjects were asked to complete the tasks as quickly and accurately as possible. Data collected included tracking data via the joystick port, EMG activity (Motion Lab Systems, Inc.) of eight muscle groups (anterior deltoid, posterior deltoid, latissimus dorsi, pectoralis major, biceps, triceps, and forearm flexor and extensor groups), and three views of video using the MU-Lab system.

### Data and statistical analysis

The data was analyzed across subjects within the same experiments. For analysis, stroke survivors were partitioned according to their Fugl-Meyer motor impairment levels into two groups: *high *function (58–66) and *low*-to-medium function (22–57).

#### EP1 and EP2 tasks data and statistical analysis

The pseudo-random sinusoidal tracking was analyzed across subjects within joystick and wheel tasks using two continuous tracking metrics from Table 2: the Percentage Time on Target (PTT) and RMSE metrics; the pseudo-random target acquisition was analyzed using discrete tracking metric defined in Table 2: the Movement Speed metric. These data have been analyzed with the special attention paid to validate **hypotheses 1 and 2**. Mean and standard deviation values are calculated and presented for control (n = 8), high function (n = 5), and low function (n = 4) groups for the joysticks and high function (n = 4) and low function (n = 3) groups for the wheel. A mixed-design repeated measure ANOVA test was used to test between group (by functional level) and within group (by device type) difference with Bonferonni test used for post-hoc analysis. A significance threshold level of p < 0.05 was used for interpretation.

#### EMG processing and analysis

While a wide variety of data were collected during the TheraJoy positioning study, the focus of analysis here is on EMG. Each EMG file passed through standard signal processing techniques including a filter to remove the average signal value and remove any signal offset, a high pass filter Butterworth filter with a corner frequency of 60 Hz to remove noise in the signal due to cardiac muscle, and an RMS low-pass filter (window length = 0.15 seconds, window overlap = 0.075 seconds). Upon completion of all tasks for a given subject the overall maximum RMS value was used to scale each EMG. All RMS data was then passed through a threshold filter and binned in one of 8 bins according to a proportion of the greatest value observed for each subject and each muscle with cutoffs at 85%, 70%, 55%, 40%, 25%, 10%, and 5%. The data have been analyzed with the special attention paid to validate **hypothesis 3. **Trends are described.

## Results

### Hypothesis 1 and 2: sensitivity of metrics across subjects and devices

**Figure 5:**
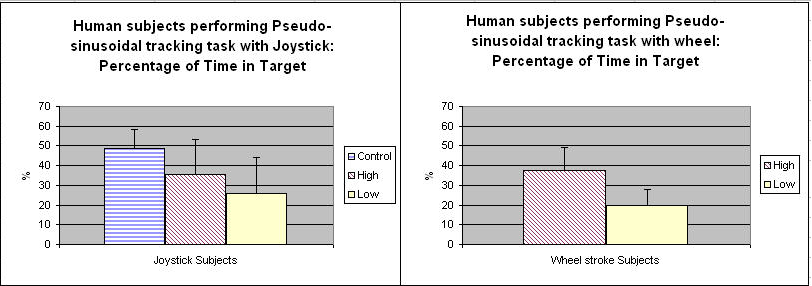
**Percentage Time on Target (PTT) for continuous tracking for CJS and TheraDrive wheel**. This figure shows PTT for continuous tracking on the conventional joystick (a) and wheel (b) for control, high function and low function groups. For joystick settings, control group PTT = 48.89 +/- 9.60, high function group PTT = 35.45 +/- 18.02, low function group PTT = 25.83 +/-18.25; for wheel settings, high function group PTT = 25.83 +/- 18.25, low function group PTT = 19.72 +/- 8.04.

Figure 5 shows Percentage Time on Target (PTT) for EP1 group using the joysticks and EP2 group using the wheel for the continuous pseudo-random sinusoidal tracking tasks. The results on the PTT show that controls and high functioning stroke subjects had a tendency to be more accurate and stable than low functioning subjects. For the between group difference, there was a significant difference between control and low function group (p < 0.01) on the joysticks, and a trend indicated difference between high function and low function group in (p < 0.1) on the wheel; the low function group had a lower PTT in both cases. For the within group difference, there was no statistical difference between joystick and wheel settings.

**Figure 6:**
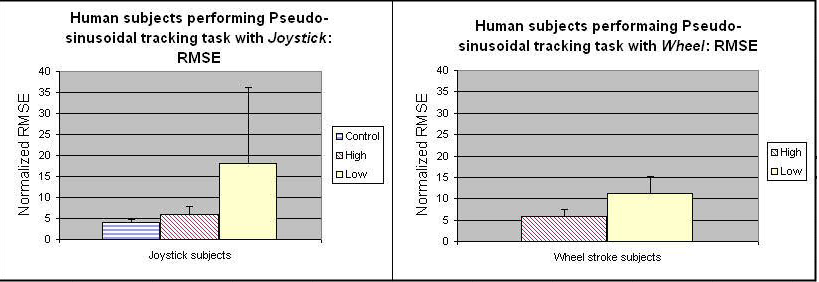
**RMSE for continuous tracking for CJS and TheraDrive wheel**. This figure shows RMSE for continuous tracking on the conventional joystick (a) and wheel (b) for control, high function and low function groups. The RMSE is normalized to percentage of the workspace. For joystick settings, control group RMSE = 3.99 +/- 0.67, high function group RMSE = 6.05 +/- 1.80, low function group RMSE = 19.05 +/-18.12; for wheel settings, high function group RMSE = 5.81 +/- 1.64, low function group RMSE = 11.31 +/- 3.86.

Figure 6 shows the normalized root mean square errors (RMSE) for the joysticks and wheel across the EP1 and EP2 groups for the continuous pseudo-random sinusoidal tracking tasks. The results on the RMSE show that controls and high functioning stroke subjects had a tendency to be more accurate than low functioning subjects. There was a significant difference between control and low function group (p < 0.01), as well as between high and low function group (p < 0.05); there was also a trend observed that low function group subjects using conventional joystick had a bigger RMSE than low function group using driving wheel. The results could suggest a possible sensitivity to device complexity; having to control 1-D versus 2-D may have made a difference in tracking performance in the low functioning subjects.

**Figure 7:**
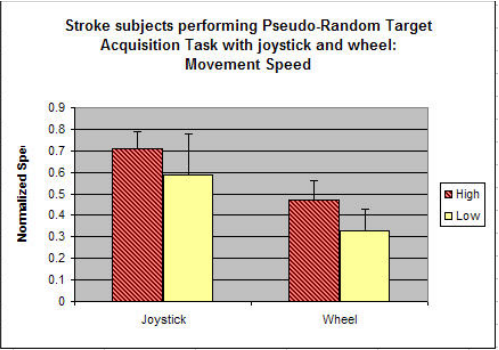
**Movement Speed for pseudo-random target acuiqisiton task across conventional joystick (EP1) and wheel (EP2) by both high and low functional group**. This figure shows Movement Speed (MS) metric for pseudo-random target acquisition for the stroke subjects using conventional joystick (EP1) and wheel (EP2). Note: For joystick settings: high functional group's MS = 0.71 +/- 0.08, low functional group's MS= 0.59 +/- 0.19; for wheel settings: high functional group's MS = 0.47 +/- 0.09, low functional group's MS= 0.33+/- 0.10;

Figure 7 shows the normalized movement speed across conventional joystick and wheel by high and low functional group in both EP1 and EP2 for the pseudo-random target acquisition task. There is a significant difference between high and low functional group (p < 0.05) and between the joystick and wheel (p < 0.01). Subjects moved the driving wheel at lower speeds in the target acquisition task; one possible reason may be to the higher inertia of the wheel.

The metrics analyzed were able to differentiate between the two levels of subjects (LOW and HIGH) suggesting their utility in detecting improvements in subjects as they recover. The metrics also differentiated across types of devices (wheel and joystick) suggesting the possible utility in using the device types to grade therapy (simple to complex movement) and in defining protocols that are tailored to the functional ability of a patient.

### Hypothesis 3: effects of device and device location on EMGs for EP3

Overall EMG results show that not only does each device target different muscle groups, but also that changing the position of the device relative to the shoulder also alters control strategies. A general tendency was for all muscles to display increased average activity from the conventional joystick (CJS) to the horizontal TheraJoy (HJS) and then again to the vertical TheraJoy (VJS).

**Figure 8:**
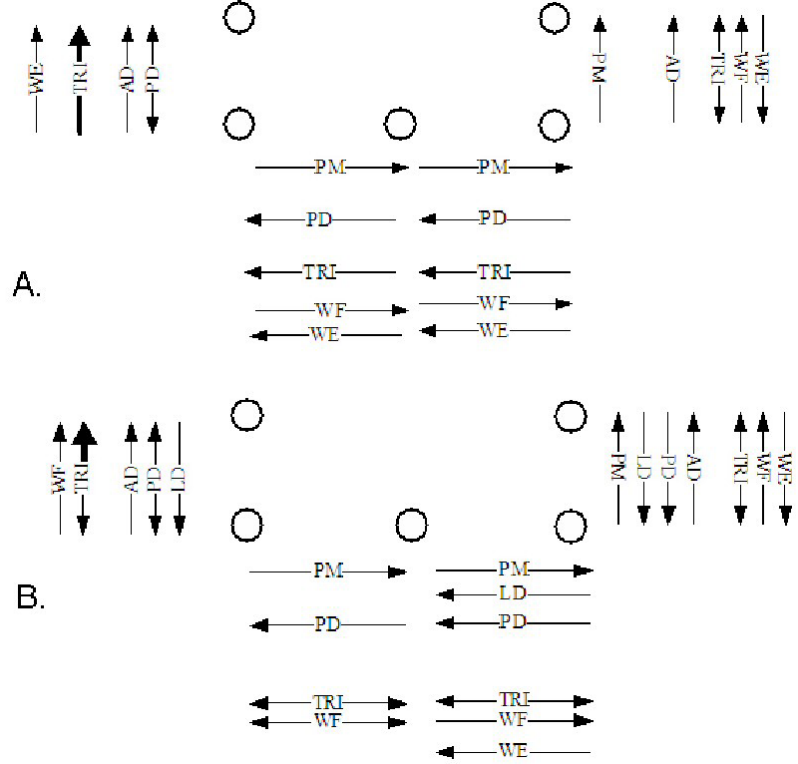
**Muscle control strategy shifts for rectangle task for HJS**. EMG representations of active muscles during the bottom half of the 3 point rectangle task while using the horizontal TheraJoy in (a) the neutral position (b) close to the body, neutral to the shoulder. Each muscle displaying average activity between either 10 and 25% or 25 and 40% of the maximum value is represented by a thin or thick arrow, respectively.

Figure 8 (A and B) displays a representation of the muscle activity observed during both clockwise and counter clockwise rotations of the lower half of the rectangle tracking pattern on HJS. Figure 8A represents muscle activity observed during the neutral position whereas 8B represents the position close to the body, neutral to the shoulder. The muscle activation pattern for the latissimus dorsi, posterior deltoid, and the triceps changed noticeably. When positioned close to the body and neutral to the shoulder, the latissimus dorsi is used as an agonist to internally rotate the arm to complete movements. When the device is closer to the body, the posterior deltoid has increased activity to complete movements by extending the shoulder. As the device is positioned further from the body, the triceps takes over and is able to extend the elbow.

In contrast to the horizontal joystick, the vertical TheraJoy (not shown) had overall increased muscle activity, especially in the anterior deltoid, latissimus dorsi and biceps. In evaluating movements in opposite directions within the same workspace, it is clear that anterior deltoid is especially necessary to hold positions where the arm is elevated at or above the shoulder height, specifically when the device is positioned across the body with a neutral position in line with the sternum. During movements in this workspace subjects spent at least 17% of each movement with medium levels of EMG activity. In contrast, during activities in the lower region of the workspace, the anterior deltoid is off during at least 49% of each movement.

These results indicate that each device uses different muscle control strategies even for the same screen task, including both the use of different muscle groups and different magnitudes of each muscle. This affects exercise prescription. For instance, to promote agonist anterior deltoid activity, the horizontal and/or vertical TheraJoy would be recommended, assuming that the patient is able to move for a reasonable range of motion within these workspaces. Therefore, our results suggest that the device type and device location are two variables that can be used to help personalize therapy to promote functional recovery of specific muscles and arm movements.

## Discussion

Our results support the potential benefit of the Robot/CAMR suite for stroke rehabilitation. The Robot/CAMR Suite provides several key variables that can be used to create a personal therapy environment. Therapists can choose the type of tracking task, the therapeutic device, the device location about the subject, and assessment metrics to match the need of the patient. Despite small data sets across the experiments analyzed, we were able to show that different device interfaces (wheel and conventional joystick) and device settings (device location in the arm workspace) significantly affect tracking and muscle performance outcomes. In addition, we demonstrated the ability to distinguish between subjects on different functional levels.

Three experiments evaluated the potential of the suite using the toolboxes and strategies outlined. We can distinguish between motor performances of subjects with different functional levels (on the same task). We found that our PTT and normalized RSME metric could detect differences in accuracy across low and control/high functioning subjects on a continuous tracking task, as well as movement speed metric for target acquisition task. Low-medium functioning stroke subjects performed significantly worse than able-bodied and high functioning stroke survivors for joysticks and close to significantly different on the wheel (Fig. 5, Fig. 6 and Fig. 7). Trends in this assessment metric were consistent across devices (Fig. 7).

We hypothesized that assessment metrics can distinguish between motor performances of the same subjects using different devices. We tested the conventional joystick (EP1) and driving wheel (EP2) in two tasks: pseudo-random sinusoidal continuous tracking and pseudo-random target acuiqisiton. Significant differences were shown by movement speed metric in target acquisition task: wheel moves much slower than joystick. The lack of difference by PTT and RMSE in continuous tracking task across device type could be due to the fact that the metrics were not sensitive enough to the device differences or that the mapping of the ROMs for the device to the task diminished the effect of the differences on motor performance. The first reason may be more likely when considered against the published results from Johnson and colleagues [16], which showed that movement time was significantly different for each device. Further investigations would be needed to exclude the second reason. This also suggests that it is important that the appropriate assessment task as well as kinematic metric should be chosen for analyzing motor performance across the device.

It is important to note that different muscles were recruited with different joysticks types and with different joysticks positions in the arm's workspace. This seems like a trivial result but the versatility of the Robot/CAMR suite rides on the fact that different devices in suite can be easily chosen and then configured to focus on training different muscles of the arm using different tracking activities. The results from EP3 showed that different devices (VJS and HJS) utilized different muscle combinations and control strategies for shoulder and elbow during tracking tasks and for a given location. Within these devices, the direction of the movement was also important with movements toward the lateral edge of the workspace increasing muscle activity. As an example, neutral positions of HJS are lead to higher posterior deltoid activity, occurring in medial regions of the workspace. The position of the device was also important and can be a useful variable to vary. Muscles such as pectoralis major, latissimus dorsi, triceps and biceps were sensitive to device position. Now, if the deltoid was our target muscle, then the HJS would be chosen of the VJS or the wheel as the main device in the suite to be used for therapy. Device type and its relative location can be successfully varied in the Robot/CAMR device suite.

Although not explicitly analyzed, feedback collected from most participants with stroke-induced impairment found the devices very enjoyable to use, and commented to investigators that although they did not frequently use a computer, if given the opportunity to use these devices their level of use may increase. The subjects enjoyed using the third party software such as Pacman (EP1) and SmartDriver (EP2) and were motivated to play to increase their game score. All were satisfied with the technology, operability and comfort of the system. Most responded positively to the questions asking if they would use the system frequently and if they would use the system in their home.

In summary, the results suggest that in the Robot/CAMR Suite, the tracking tasks, the devices and the device location about the user are variables that can be used to create a stroke therapy environment that is tailored to the user's needs. The challenge here is in identifying the optimum combination for subjects and creating a seamless mapping between these variables and the user's disability and therapeutic needs. Further research is needed.

The main limitations of our study were in the small sample size and that our subject population across devices weren't always the same. In addition, our stroke population was polarized in that we did not fully span the disability workspace. Despite these limitations, our results suggest that the concept of a distributed suite of systems has great potential for personalizing stroke rehabilitation. All the devices combined would create a versatile and flexible framework for therapy. A larger longitudinal study is still needed to evaluate these systems in the home or in an under-supervised environment.

## Conclusion

There is a need to improve the cost-to-benefit ratio of robot-assisted therapy strategies and their effectiveness for stroke therapy in home environments characterized by the low supervision by clinical experts, low extrinsic motivation as well as low cost requirement. Our distributed device approach to this problem consisted of an integrated suite of low-cost robotic/computer-assistive technologies driven by a novel software framework. Our strategy for personalizing therapy, sustaining motivation and ensuring adequate assessment was presented. We evaluated the potential of the concept via three studies. The results support the fact that the choice of a task, metric, the device and its location in the workspace with respect to the user influence the performance outcomes and therefore can be used to personalize therapy to fit the therapeutic needs of the given client. It also supports the use of low-cost mass-marketed devices in goal-directed performance assessment: the results demonstrated the ability of our platform to distinguish between low function and control/high function subjects. These outcomes combined with preliminary receptivity of stroke subjects and therapists to the systems suggest the need for further study. A larger longitudinal study is still needed to evaluate these systems in the home or under-supervised environment and to determine how well these results can be generalized.

## Competing interests

The author(s) declare that they have no competing interests.

## Authors' contributions

LMJ, and XF were involved in all stages of subject recruitment and data acquisition. MJJ and XF were the primary composers of the manuscript with major contributions LMJ and JM. JM generated the initial concept for TheraJoy studies and oversaw their progress while MJJ generated the initial concepts for TheraDrive studies and oversaw their progress. LMJ and JM designed and built the TheraJoy hardware. MJJ designed and built the TheraDrive hardware with the assistance of colleagues at the Clement J. Zablocki VA. XF and JM designed and built the software used for training with assessment metrics with input LMJ, and MJJ. All authors contributed significantly to the intellectual content of the manuscript and have given final approval of the version to be published.
